# Better Than Vaccination If True

**Published:** 1883-06

**Authors:** 


### BETTER THAN VACCINATION IF TRUE.

The following excerpt from a New Orleans journal, offers something brand new, and if true in all the puarticulars it claims, it is a greater discovery than that of Sir William Jenner.

When Dr. Tebault has demonstrated both the truthfulness and philosophy of his novel process with the clearness and success of Jenner, he may not find a monarch to knight him; but something far better, the applause of a grateful world:

Two of the members of the special committee of the State Board of Health (on contagious diseases) were unavoidably prevented yesterday evening from meeting Dr. C. H. Tebault, at his office, to discuss his new process of modified inoculation. Dr. Formento, the chairman of the committee, was present, and a lengthy conference between the doctors followed. Dr. Tebault said that for the past 20 years he had been performing these operations of modified inoculation, and in no case had it ever failed on his hands. He had operated with success upon small-pox cases who had recovered from the disease, within six months of such recovery, and had in such cases repeatedly tried the best vaccine, but without result. His reasons for reccommending the practice of his modified inoculation are these:

1. It never fails, and it acts more promptly than vaccination.

2. All his experience assures him that persons so operated on do not communicate small-pox by contact, acting in this respect precisely as vaccination.

3. Physicians can always procure it from a known healthy source, and can always have it on the appearance of the first case in our midst.

4 The presence of this loathsome and dreaded diseases by this operation can be converted from a cause of infection into a means of positive protection to all exposed.

5. Inoculation, thus modified, procures effects as uniform as does vaccination, with the advantage that it is always successful; whereas vaccination has frequently to be repeated several times before it succeeds, thus losing valuable time, when every moment may be important.

6. Modified inoculation will succeed, it matters not how often the person operated on has been successfully vaccinated.

The operation consists in taking the first vesicle that presents itself, and admixing the lymph so secured from this vesicle with the cow's milk in the proportion of one drop of the lymph to three or five of milk, according to the age of the subject to be operated on, and applying a small quantity of this to the arm in the same manner that vaccine is applied.

